# Topics of Public Interest

**Published:** 1918-08

**Authors:** 


### TOPICS OF PUBLIC INTEREST

**Casualties on the Western Front** are said by Maesfield to average 10 a minute (about equally divided between the contestants)—representing about one killed and one permanently disabled or captured for each side, or a total of 5 million for both sides, or definitive losses of 1 million for each side.

### Influence of War on Migration.

| | Immigration of Aliens | Non-Immigrants arriving | Total arrivals | Total departures Emigrants and other |
|---|---|---|---|---|
| 1913 ..... | 1,387,318 | 229,585 | 1,616,903 | 283,378=1,033,525 |
| 1914 omitted as not typic. | | | | |
| 1915 ..... | 258,678 | 68,963 | 327,641 | 284,636= 43,005 |
| 1916 ..... | 355,587 | 72,904 | 428,491 | 164,784= 263,707 |
| 1917 ..... | 152,959 | 58,926 | 211,885 | 131,183= 80,702 |
| | | | | net arrivals. |

In estimates of growth of population, it should be remembered that, approximately, our increase has been about 20% per decade, which does not mean quite 2% per annum, any more than it would for a problem in compound interest. It should also be remembered that about half of this increase has been due to immigration. The present census decade will have been subject to the 20% increase for just about 1/3 of the period and to a 10% increase for the remaining 2/3, as it seems unlikely that any appreciable immigration will be possible before 1920, not to mention our military losses which will probably not be very high. We anticipate, therefore that various vital statistics carried by estimates, will prove to have considerable inaccuracies, when checked up at the next census year.

**Belligerent and Neutral Population of the World.** In round numbers, the Central allies have 160, millions; the Entente Allies 1,360, millions, neutrals number 120, millions, the total population being 1,600, millions. It must be admitted that many of the subjects of the Entente Allies are savage or barbarian, in a cultural, not a kultural sense and that many nations will not take an active part in the fight. But with all allowance, the only logical conclusions to the outcome of the war is the optimistic one.

**The U. S. Public Health Service** (for the first time, we believe) announces examinations for women for the position of acting assistant surgeon, corresponding approximately to the commission as first lieutenant in the Army Medical Corps.

---

**Cremation.** The first English society was founded in 1874, the first crematory in the U. S. 2 years later. From 1876 to 1884 only 28 cremations occurred in this country. 58 the next year, 1005 in 1895 and from this date, there has been a very steady rise to 12,767 in 1915, not quite 1% of all deaths. More recent statistics are not yet available.

---

**Precocious Venereal Disease.** E. Finger, Wiener Med. Woch., Mch. 9, states that since the war began, the ratio per 1000 of young subjects showing venereal disease has increased from 1 to 8 for boys aged 15; from 3 to 9 for 16; from 11 to 26 for 17; from 27 to 68 for 18; aggregates from 42 to 111. For girls, as might be expected, the increase has been proportionately less though absolutely more: 12-16 at age 15; 22-40 at 16; 33-66 at 17; 62-91 at 18; aggregates from 129 to 213. As previously noted, juvenile crime in general has increased in Germany on account of the war and probably the same has occurred in other countries. This should be a caution to us.

---

**Horse Meat.** About 1000 butcher shops in Paris deal exclusively in horse and ass meat, the average sales being 4-5 carcasses a week for each.

---

**Medical and Other Incomes.** In 1917, 61% of engineers (civil, mining, etc.) reported incomes over $3,000. Other percentages were: insurance agents, 28; stock and bond brokers and other brokers, 20; lawyers and judges, 19; mine owners and operators, 18; manufacturers and manufacturers, 10; architects, 8.5; army and navy officers and the medical profession (20,348 reporting) 7; editors, authors and reporters 6.5; commercial travelers and real estate dealers, merchants, etc., 4.5; saloon keepers 1.9; clergymen 1.4; actors and musicians 0.55; teachers 0.47; farmers 0.24. It will be noted that the percentages are merely for those making returns at all. Thus, if the returns are honestly made, the actual percentage of physicians earning $3000 or more, net, is 1%.

---

**A New Collar Insignia** has been adopted for the Army Air Service. It consists of a pair of horizontal bronze wings, similar in shape to a colonel's eagle's wings, with a silver two-blade propeller placed vertically on the wings. It will be worn by officers and enlisted men of the Department of

Military Aeronautics and the Bureau of Aircraft Production, and it takes the place of the torch and crossed flags worn by them when under the Signal Corps. The hat card for enlisted men of these two branches of the air service will be green and black.

---

**Persons** desiring to send money to prisoners of war in territory controlled by the Central Powers should send it through the Bureau of Prisoner Relief of the American Red Cross, Washington. This society has been granted a license for such business by the War Trade Board.

---

**A Special Cap,** officially known as the "over-seas cap," is now being worn by the soldiers of the American Expeditionary Forces. It matches the uniform in color, is round and has no brim or peak. The crown is made very low and made so that when not in use, it can be folded and carried in the pocket. It is made but not issued in this country.

---

**A Military Postal Express Service** has been established. This service will receive from the civil postal authorities all mail arriving in France for the American Expeditionary Forces and distribute it.

---

**The Public Health Service** urges all physicians to report all communicable diseases, no matter how small, as a protection to the selected and enlisted men who are traveling and who could carry the germs back to camp.

---

**A Problem for Antivivisectionists.** Laboratory animals are now somewhat difficult to get. They are needed to save the lives of soldiers. For example, up to date, we have probably lost more men by pneumonia than by hostile weapons and missiles of all kinds. One type of pneumonia is very successfully treated by a serum, while the other three types are not. It is obviously necessary to determine which type a man has in order to apply the proper treatment, and the type is determined mainly by culture in animals. This is admittedly somewhat hard on the animal though the injection is not painful to a high degree, if indeed it can be described as more than uncomfortable. The suggestion comes from the laboratory men themselves that it would save a large expense and inconvenience and perhaps human lives by saving time, if, instead of determining the type of penumonia by animal cultures, it could be done by some form of test tube experiment which would not involve animal life at all. If the antivivisectionists are sincere, here is a chance for them to show their humanity both to man and the lower animals in a way that is also patriotic and that will help win the war just as directly as the invention of an armor that will prevent a certain number of fatalities by rifle balls. Such an accomplishment would be of great abstract scientific value and it would, in its general principles and probably in its practical developments apply to many other diseases. Suppose the antivivisectionists devoted part of their funds to finding substitutes for animal experiments of this kind instead of putting it up to the medical profession to spare the animals and let human beings die.

---

**Venereal Diseases—N. Y. State Law.** According to the new law, effective April 17, only licensed physicians may treat or prescribe for such diseases; pharmacists filling prescriptions are to retain them, no copy being given to the patient and no prescription to be refilled.

---

**Infant Mortality for N. Y. City.** In 1907—the mortality was 135.8 per 1000 births, in 1917, the minimum was reached —88.8.

---

**The Attendance at the 1918 A. M. A. Meeting** was 5,553, the largest in the history of the organization except the previous Chicago meeting of 1908 when the registration was about 800 more. This attendance is remarkable in view of the large number of physicians who have entered military service.

---

**Military Orders.** Men newly commissioned in the M. R. C. do not generally understand the importance of preserving and keeping instantly available for reference, the orders, telegraphic or otherwise, directing travel, duty, etc.

---

**Statistics of Troops Abroad.** It seems that the U. S. has successfully camouflaged the movement of troops by telling the truth and allowing outsiders to make estimates on various statistics, from sick reports, etc., for which just enough actual numbers were given to mislead spies and the innocently curious. Up to Aug. 1, 1917, at which date "those who claimed to know" estimated at least 50,000, 26,967 had sailed. Up to Nov. 1, when the press was allowed to state that upwards of 100,000 had sailed and when it was semi-publicly announced by the wise ones that about 400,000 had crossed, the actual number was 116,072. Up to Feb. 1, when calculations by difference between the totals of all three military organizations and the numbers publicly shown to be in this country, made it appear that somewhere between 600,000 and 700,000 had crossed, the actual number was 234,704. Up to May 1, the number, as announced, was just about 500,000 and, up to July 1, the total is 1,019,113.

---

**Lynchings.** Robert R. Moton, Principal of Tuskegee Institute, states that 35 have occurred in the first six months of 1918, similar periods of the last two years being 14 and 35, respectively. 34 of the persons lynched were colored, including 3 women. 8 lynchings were for rape. If no general appeal for respect of law avails, it ought to be realized that there is, during the war, a special patriotic reason for refraining from lynchings, more particularly when lynchings are excused as due to indignation against unpatriotic deeds. Every lynching in the U. S. affords our enemy an excuse for lynching in Europe.

---

**Railroad Accidents.** 206,000 persons were injured and 1000 killed in 1917, a reduction of about 20% from 1916.

---

**The Possibility of Census Camouflage.** Various statistics, partly emanating from Germany, partly estimated on the general basis of an average equality of military losses on the two sides, partly based on number of divisions, agree pretty closely with total definitive German losses of about 4 million, leaving about 3 million of the original 10% militia strength, the draft of youths just about balancing non-military losses. It has been suggested that Germany had 90 instead of about 67 millions population at the outbreak of the war. A difference of this degree obviously could not have been concealed between any two decennial censuses and it would produce inconsistencies in vital statistics, even in food consumption and industrial statistics generally. Such a proportionate discrepancy would undoubtedly have been detected if applied to small communities, especially near the border and with the careful watch which the various European powers have kept on one another for many years, it is questionable whether a census camouflage even begun many years ago, could have been successfully carried out. However, the question is an interesting one, academically, aside from its bearing on the possible duration of the war.

---

**Trench Fever.** From various reports and original articles, the following facts are gathered. No specific germ has been isolated nor is there evidence that a known germ causes the symptoms. But the disease seems to be an infection, transmitted by louse bite as 12 of 22 healthy volunteers became infected and 15 of 16 into whom direct inoculations of virus were made. 4 bitten by lice imported from England remained free of disease. The incubation period was 5-20 days by direct inoculation; 15-35 days by louse bite.

---

**Sugar.** 1,600,000 tons are estimated as available for the last half of 1918 for the U. S.—about 15 grams per meal per person, something like half the average consumption, a third of the ordinary consumption and a fifth of that of persons who maintain an excessive sugar ingestion without developing manifest disease.

---

**The Woman Physician in Moving Pictures.** Mme. Petrova, in the Inner Life combines the beauty and wealth of a prominent woman physician of one eastern city with the bacteriologic skill (somewhat exaggerated in therapeutic results, of course) of another woman physician.

---

**What Will Win the War.** We agree with the slogans that food, or wheat or some other food in particular, or thrift, or investment in Liberty Bonds and War Savings Stamps, or laughter, or prayer, or garbage saving or a host of other things will win the war. But we would qualify this agreement with the belief that the real thing that will win the war will be fighting in the front lines. Probably medical, surgical and sanitary skill represent the next most direct factor. For one qualified along these lines, his value is obviously far greater as an indirect than as a direct fighter. The country needs more physicians and while, in one way or another, it can use men of all ages, what is most needed is fairly young men who will volunteer for service in the M. R. C. The reserve supply now consists of a comparatively small number, not much over a thousand, and more than a third of these are over 40 or physically disqualified for service at the front.

---

**The Government** has arranged the Base Hospitals of this country as follows: General Hospital No. 4, Fort Porter, N. Y. will care for the insane; No. 7, Roland Park, Baltimore, will care for the blind and deaf; No. 13, Dansville, N. Y., will care for epileptics and neurotics; the Walter Reed Hospital at Tacpma Park, Washington, D. C. and the Letterman General Hospital, San Francisco, will make special provision for amputation work. Other hospitals chosen are General Hospital No. 2, Ft. McHenry, Mo.; No. 3, Cononio, N. J.; No. 6, Ft. McPherson, Ga.; No. 9, Lakewood, N. J.; No. 14, Ft. Oglethorpe, Ga.; Army and Navy General Hospital, Hot

Springs, Ark.; Base Hospital, Des Moines, Ia.; Base Hospital, Ft. Riley, Kan., and Base Hospital, Ft. Sam Houston, Tex. Individual hospitals from this group will be equipped for special work in cardivascular diseases, tuberculosis, neurological and other head surgery cases, for speech defects, general medicine, and other specialties.

---

**Packages for Overseas Delivery** not accepted unless accompanied by a written request from the soldier and approved by a major or higher commanding officer. Persons connected with the Red Cross and other organizations in France, must make a request for articles in a similar manner, the approval of an executive officer of the organization being necessary in such case.

---

**Dr. Biggs, State Commissioner of Health** urges that people inquire as to the purity of the water and milk supply, the safe disposal of refuse and other sanitary conditions that affect the health, before choosing a vacation spot.

---

**The Medical Department in France** had mobile apparatus to provide fresh water for the soldiers. The trains and miniature waterworks, which chemically treat, filter and sterilize water, making it fit for consumption. A number of purification units with attached motor-tank trucks constitute a train. Each unit is a complete filtration plant including laboratory. Arriving at a stream it sets hose into the water and pumps the water through a pressure tank. Before the water passes through a sand filter it is treated chemically to rid it of disease germs. The pure water is pumped into tanks mounted on trucks, which carry the water to the soldiers. Each mobile water unit carries an expert chemist, bacteriologist, and pump man. There is a complete laboratory in the front of the machine for the testing of the water. Tests are made every two hours or more often when it is thought necessary. The water is lifted into the filter by a gasoline pump engine, and a complete supply of extra pipes and tools are carried so that all repairs, either from accident or shell fire can be made on the spot. The trucks are equipped with electric lights so that the work can be carried on at night.

---

**Women Health Officers,** whose duty will be to see that the thousands of women workers in munitions plants are kept healthy and their output of war materials thus maintained at the peak of production, are to be trained under the direction of the women's division of the industrial service section of the Army Ordnance Department, The women who are taking the course are college graduates, or women of equivalent technical education. Almost all have had experience in dealing with working women.

---

**Secretary Baker** now prohibits the serving of liquors to officers and men of the Army anywhere in the U. S., even within private homes.

